# Divergent transcriptomic responses underlying the ranaviruses-amphibian interaction processes on interspecies infection of Chinese giant salamander

**DOI:** 10.1186/s12864-018-4596-y

**Published:** 2018-03-20

**Authors:** Fei Ke, Jian-Fang Gui, Zhong-Yuan Chen, Tao Li, Cun-Ke Lei, Zi-Hao Wang, Qi-Ya Zhang

**Affiliations:** State Key Laboratory of Freshwater Ecology and Biotechnology, Institute of Hydrobiology, Chinese Academy of Sciences, Wuhan, 430072 China

**Keywords:** Interspecies infection, Ranavirus, Amphibian, Transcriptome response, Chinese giant salamander, Virus-host interactions, Immune pathway

## Abstract

**Background:**

Ranaviruses (family *Iridoviridae*, nucleocytoplasmic large DNA viruses) have been reported as promiscuous pathogens of cold-blooded vertebrates. *Rana grylio* virus (RGV, a ranavirus), from diseased frog *Rana grylio* with a genome of 105.79 kb and *Andrias davidianus* ranavirus (ADRV), from diseased Chinese giant salamander (CGS) with a genome of 106.73 kb, contains 99% homologous genes.

**Results:**

To uncover the differences in virus replication and host responses under interspecies infection, we analyzed transcriptomes of CGS challenged with RGV and ADRV in different time points (1d, 7d) for the first time. A total of 128,533 unigenes were obtained from 820,858,128 clean reads. Transcriptome analysis revealed stronger gene expression of RGV than ADRV at 1 d post infection (dpi), which was supported by infection *in vitro*. RGV replicated faster and had higher titers than ADRV in cultured CGS cell line. RT-qPCR revealed the RGV genes including the immediate early gene (*RGV-89R*) had higher expression level than that of ADRV at 1 dpi. It further verified the acute infection of RGV in interspecies infection.

The number of differentially expressed genes and enriched pathways from RGV were lower than that from ADRV, which reflected the variant host responses at transcriptional level. No obvious changes of key components in pathway “Antigen processing and presentation” were detected for RGV at 1 dpi. Contrarily, ADRV infection down-regulated the expression levels of MHC I and CD8. The divergent host immune responses revealed the differences between interspecies and natural infection, which may resulted in different fates of the two viruses. Altogether, these results revealed the differences in transcriptome responses among ranavirus interspecies infection of amphibian and new insights in DNA virus-host interactions in interspecies infection.

**Conclusion:**

The DNA virus (RGV) not only expressed self-genes and replicated quickly after entry into host under interspecies infection, but also avoided the over-activation of host responses. The strategy could gain time for the survival of interspecies pathogen, and may provide opportunity for its adaptive evolution and interspecies transmission.

**Electronic supplementary material:**

The online version of this article (10.1186/s12864-018-4596-y) contains supplementary material, which is available to authorized users.

## Background

Several emerging and threatening viruses are concerned with interspecies infection between human beings and lower vertebrates or invertebrates, such as Influenza A virus, Zika virus, Ebola virus, and so on. Most of them are RNA virus [1–3]. Numerous advances on interspecies infection that several species were infected by the same virus have been gained until now [4–7]. Besides this, several viruses in lower vertebrates and invertebrates also had a wide range of hosts, such as viruses in the family *Iridoviridae*, *Nodaviridae*, and *Rhabdoviridae* [8, 9]. Wide host range increased the threat caused by the virus and the possibility of interspecies infection.

Aquaculture has become one of the fastest and most efficient agricultural production industries in the world over the last three decades [10]. However, viral diseases have hampered its development [11, 12]. As a genus of the family *Iridoviridae,* viruses in *Ranavirus* have icosahedral capsids and double-stranded, nucleocytoplasmic large DNA genomes [13, 14]. These viruses have been reported as promiscuous pathogens of cold-blooded vertebrates [15] and have been isolated from different aquaculture animals including fish [16–18], amphibians [19–21], and reptiles [22, 23]. Currently ranaviruses constitute the majority of iridoviruses [13]. Many of them are highly pathogenic and represent great threat to cultured and wild lower vertebrates [18]. Sequence determination and bioinformatic analysis reveal several recent host shifts among ranaviruses [24, 25]. It has been reported that frog virus 3 (FV3, a ranavirus) or FV3-like ranavirus could infect heterologous species [26, 27].

Our lab has focused on ranaviruses for a long time, including virus isolation, genome sequencing, functional gene identification, and so on [28–35]. Among them, two ranaviruses were isolated. One is *Rana grylio* virus (RGV) which was isolated from diseased pig frog *Rana grylio* (*Anura* amphibian) [36]. The other is *Andrias davidianus* ranavirus (ADRV) which was isolated from diseased Chinese giant salamander *Andrias davidianus* (CGS hereafter, urodele amphibian) [32]. Complete sequences of the two ranaviruses have been determined [31, 32]. The genome size of RGV is 105.79 kb in length with 106 predicted genes, and the ADRV genome is 106.73 kb in size with 101 predicted genes. RGV and ADRV had a close relationship by phylogenetic analysis. Moreover, their genomes have high colinearity based on sequence comparisons. ADRV contains 99% homologous genes with RGV [32].

**Fig. 1:**
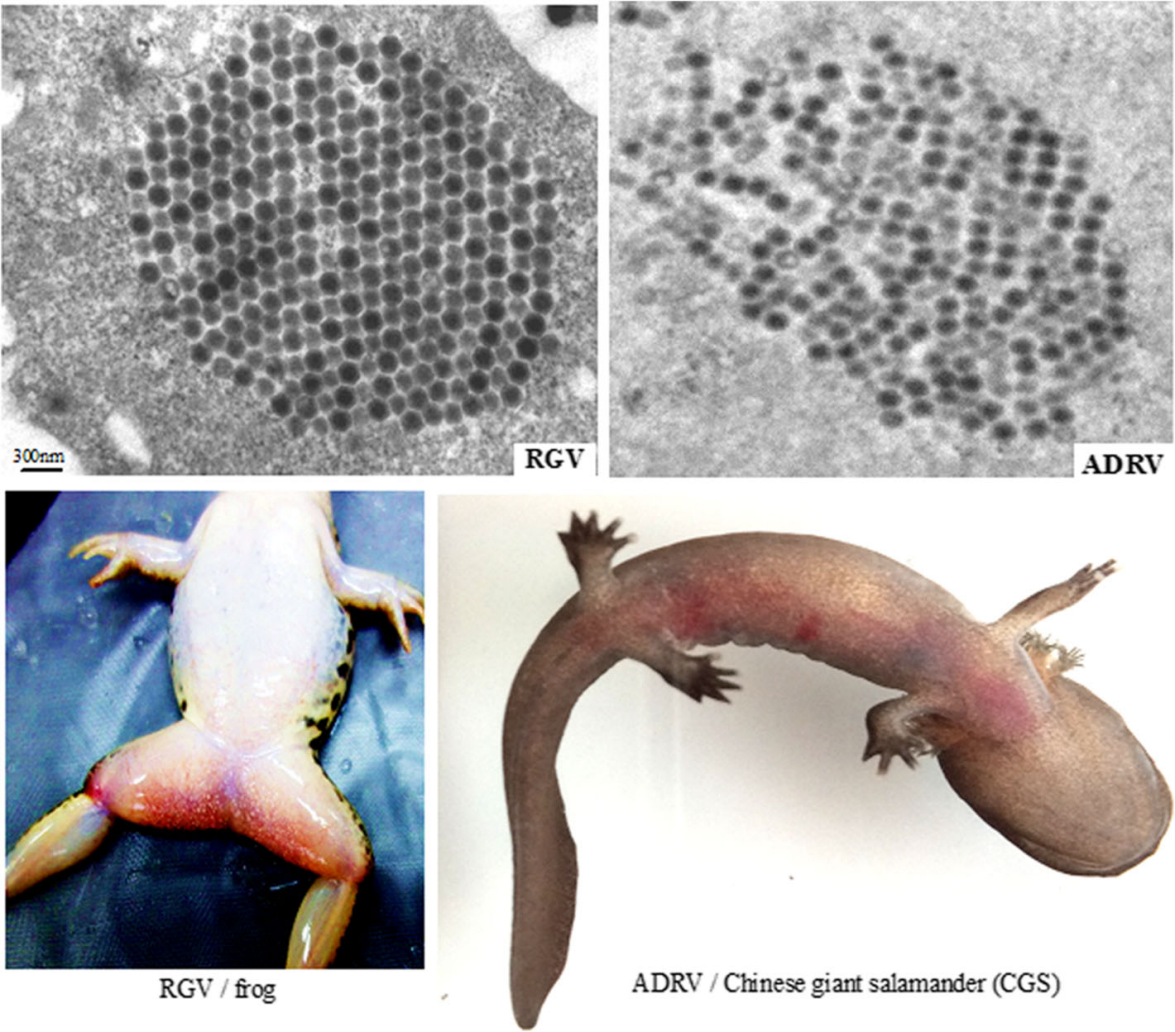
Morphology and infection of RGV and ADRV. The ultrastructure of the viruses (RGV, ADRV). And the diseased animals were infected by their respective natural pathogen (RGV/frog, ADRV/Chinese giant salamander (CGS))

The CGS is the largest extant amphibian species and known as a living fossil from 350 million years ago [37]. Although the wild population has been considered as endangered species, it has been farmed in China for scientific conservation and economic use. The two viruses (RGV and ADRV) infection in their natural host both caused systemic hemorrhage and intracellular virus particles with crystalline aggregation (Fig. 1). RGV can replicate and cause cytopathic effects in cultured CGS thymus cell (GSTC) line which was the first established cell line of CGS [38, 39]. The two viruses with high similarity provided us useful materials to investigate the interspecies infection of ranaviruses. On the basis of the above, RGV, a non-natural pathogen, was used to infect CGS as interspecies infection in this study. Simultaneously, ADRV, a natural pathogen, was also used to infect CGS. Their replication and the host responses were then analyzed with 15 transcriptome libraries.

## Results

### Differences in expression of virus genes between interspecies and natural infection

A total of 820,858,128 cleaned reads were obtained from 15 transcriptome libraries (more than 150 million reads per sample) covering RGV-1d, RGV-7d, ADRV-1d and ADRV-7d groups. The data quality evaluation with more than 94% in Q30 of each sample proved its high quality. The detailed number of cleaned reads was shown in Table 1.

**Table 1:** Summary of the sequence data of CGS transcriptome, number of virus gene with paired reads, and significantly enriched immune pathways from different treatments

Sample treatment		CGS			Number of virus genes with paired reads	Significantly enriched immune pathways						
		Number of cleaned reads	Number of Unigenes	Number of DEGs (compared to Control)		HCL	TLR	CCC	APP	NKC	III	RIG
1	Control	51,382,240	70,307									
2	Control	56,930,824	74,222									
3	Control	48,322,278	72,107									
	Control-total	156,635,342	92,642									
4	RGV-1d	54,425,646	72,588	557 (501↑, 56↓)	95	+	+					
5	RGV-1d	58,594,944	75,232									
6	RGV-1d	63,939,152	76,704									
	RGV-1d-total	176,959,742	97,513									
7	RGV-7d	49,123,866	67,404	1852 (933↑, 919↓)	16	+			+	+		
8	RGV-7d	52,736,612	73,301									
9	RGV-7d	50,018,476	70,402									
	RGV-7d-total	151,878,954	92,198									
10	ADRV-1d	56,001,870	68,499	1013 (763↑, 250↓)	29	+	+	+	+			
11	ADRV-1d	47,824,738	67,468									
12	ADRV-1d	66,289,046	69,480									
	ADRV-1d-total	170,115,654	87,131									
13	ADRV-7d	59,093,102	67,797	3880 (2694↑, 1186↓)	93	+	+	+		+	+	+
14	ADRV-7d	56,800,712	77,340									
15	ADRV-7d	49,374,622	73,957									
	ADRV-7d-total	165,268,436	95,375									
	Total	820,858,128	128,533									

*HCL* hematopoietic cell lineage, *TLR* toll-like receptor signaling pathway, *CCC* complement and coagulation cascades, *APP* antigen processing and presentation, *NKC* natural killer cell mediated cytotoxicity, *III* intestinal immune network for IgA production, *RIG* rIG-I-like receptor signaling pathway

“↑” and “↓” indicates up- and down-regulate respectively. “+” means significantly enriched

**Fig. 2:**
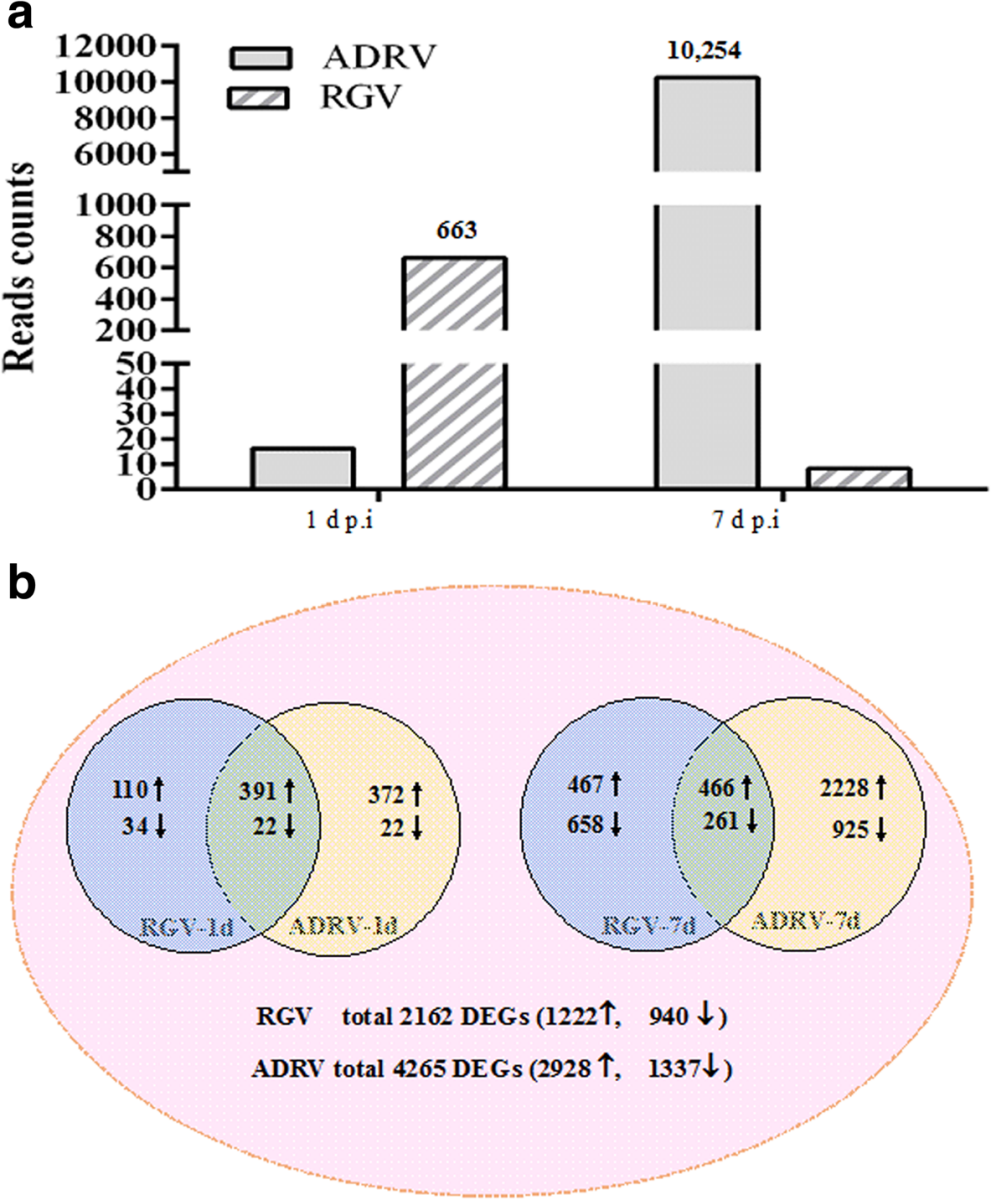
Expression of virus genes and number of DEGs (compared with control) in CGS. **a** number of paired reads covering genes of ADRV and RGV counted by featureCounts software. The Y-axes means the number of paired reads related to the genes. **b** Venn diagram of DEGs at 1 dpi or 7 dpi. Each circle represents a comparison that indicated in the cycle. Up- and down-regulated DEGs are indicated by “↑” and “↓”, respectively. Overlap region of two cycles represents DEGs in common

A portion of the cleaned reads were successfully mapped to the RGV (NCBI accession number: JQ654586) and ADRV (KC865735) genome. The number of the cleaned reads mapped to virus genome ranged from 460 to 53,720. Of them, the paired reads located in open reading frames (covered virus gene) were selected for evaluating the relative gene expression. The average number of paired reads covering virus gene was 663 for RGV-1d and 16 for ADRV-1d, while the number was 8 for RGV-7d and 10,254 for ADRV-7d (Fig. 2a). On the whole, viral reads were detected in all virus-treated groups. But the number of reads from RGV was less than that from ADRV. The number of reads from RGV-1d was more than that from RGV-7d and ADRV-1d, while the number of reads from ADRV-1d was less than that from ADRV-7d. 95 genes of RGV (106 predicted genes in all) and 29 genes of ADRV (101 predicted genes in all) had corresponding paired reads at 1 dpi, while 16 genes of RGV and 93 genes of ADRV were counted in samples at 7 dpi (Table 1). Most of the viral genes had corresponding paired reads in the libraries except 10 genes for RGV and 8 genes for ADRV. Detailed information of the paired reads was collected in Additional file 1: Table S1 and S2. The FPKM values of virus genes were also calculated based on the paired reads. The results showed a similar tendency as revealed by comparison of the number of paired reads.

### Diversity of host genes between interspecies and natural infection

128,533 unigenes covering the 15 libraries were obtained by de novo assembly (Table 1). The number of unigenes was 92,642, 97,513, 92,198, 87,131, and 95,375 for Control, RGV-1d, RGV-7d, ADRV-1d, and ADRV-7d, respectively. The N50 and average length of unigenes were 1567 and 881 bp respectively. Sequence length distribution of the unigenes was shown in Additional file 2: Figure S1.

A total of 2162 DEGs (1222 up-regulated and 940 down-regulated) in RGV treatments and 4265 DEGs (2928 up-regulated and 1337 down-regulated) in ADRV treatments were identified when compared with control. Among them, 557 DEGs (501 up-regulated) were identified in RGV-1d, meanwhile 1013 DEGs (763 up-regulated) were found in ADRV-1d. They shared 391 up-regulated and 22 down-regulated DEGs. RGV-7d possessed 1852 DEGs (933 up-regulated), while ADRV-7d possessed 3880 DEGs (2694 up-regulated), with 466 up and 261 down DEGs in common (Fig. 2b).

Eight of the top 10 up-regulated DEGs were found identical between RGV-1d and ADRV-1d, such as homologues of proteasome subunit beta type-8, serine protease, heat shock 70 kDa protein, and ATP-binding cassette sub-famlily B. Only 2 common DEGs (unknown function) were found in the top 10 down-regulated DEGs between the two groups (Additional file 3: Table S3). For the top 10 DEGs between RGV-7d and ADRV-7d, 3 up- and 2 down-regulated DEGs were identified as common DEGs respectively (Additional file 3: Table S4).

### RGV replicated faster than ADRV *in vitro*

**Fig. 3:**
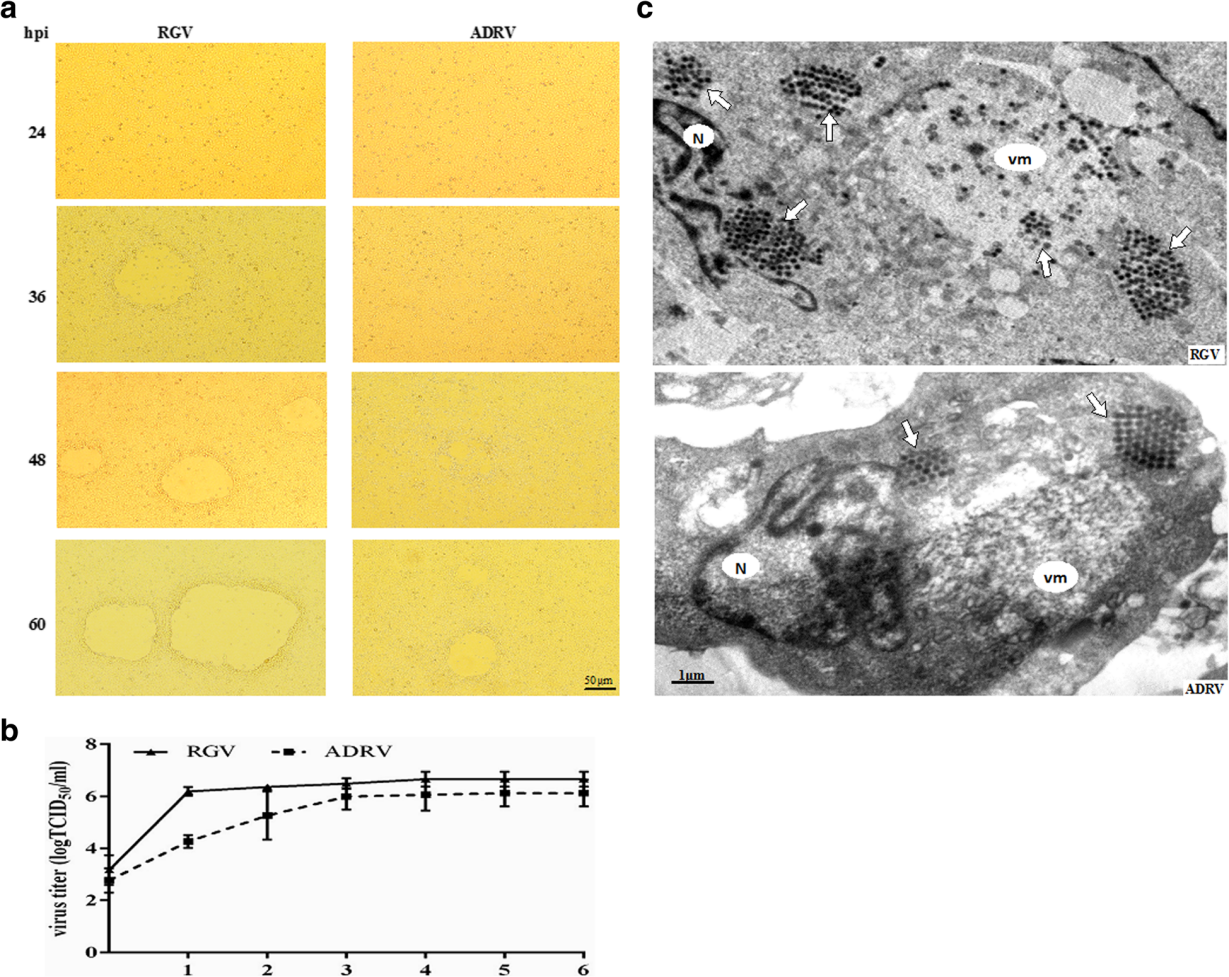
Replication of RGV and ADRV in GSTC cells. **a** the cytopathic effect (CPE) induced by RGV and ADRV in GSTC cells at different time points. **b** the one-step growth curves of RGV and ADRV in GSTC cells. The maximal titer of RGV is 10^6.9^ TCID50/mL and that of ADRV is 10^6.1^ TCID50/mL. **c** Ultrastructural observation of RGV and ADRV infected GSTC cells. The intracellular virus particles with crystalline aggregation were shown

The replication and the process of cytopathic effect (CPE) were examined in cultured GSTC cells. Both the two viruses replicated successfully in the cells (Fig. 3a). The obvious CPE caused by RGV appeared at 36 h post infection (hpi), while the CPE induced by ADRV was obvious at 60 hpi. The one-step growth curves of the two viruses was then determined. Results showed that the titer of RGV was significantly more than that of ADRV at 1 day post infection. After 4 days post infection, the growth curves of the two viruses both showed a nearly horizontal level, but the maximal titer of RGV (10^6.9^ TCID_50_/mL) was more than that of ADRV (10^6.1^ TCID_50_/mL) (Fig. 3b). The ultrastructural observation showed the intracellular virus particles with crystalline aggregation in GSTC cells for the two viruses (Fig. 3c). These results revealed that the growth of RGV was faster than ADRV.

### Host responses between interspecies and natural infection

**Fig. 4:**
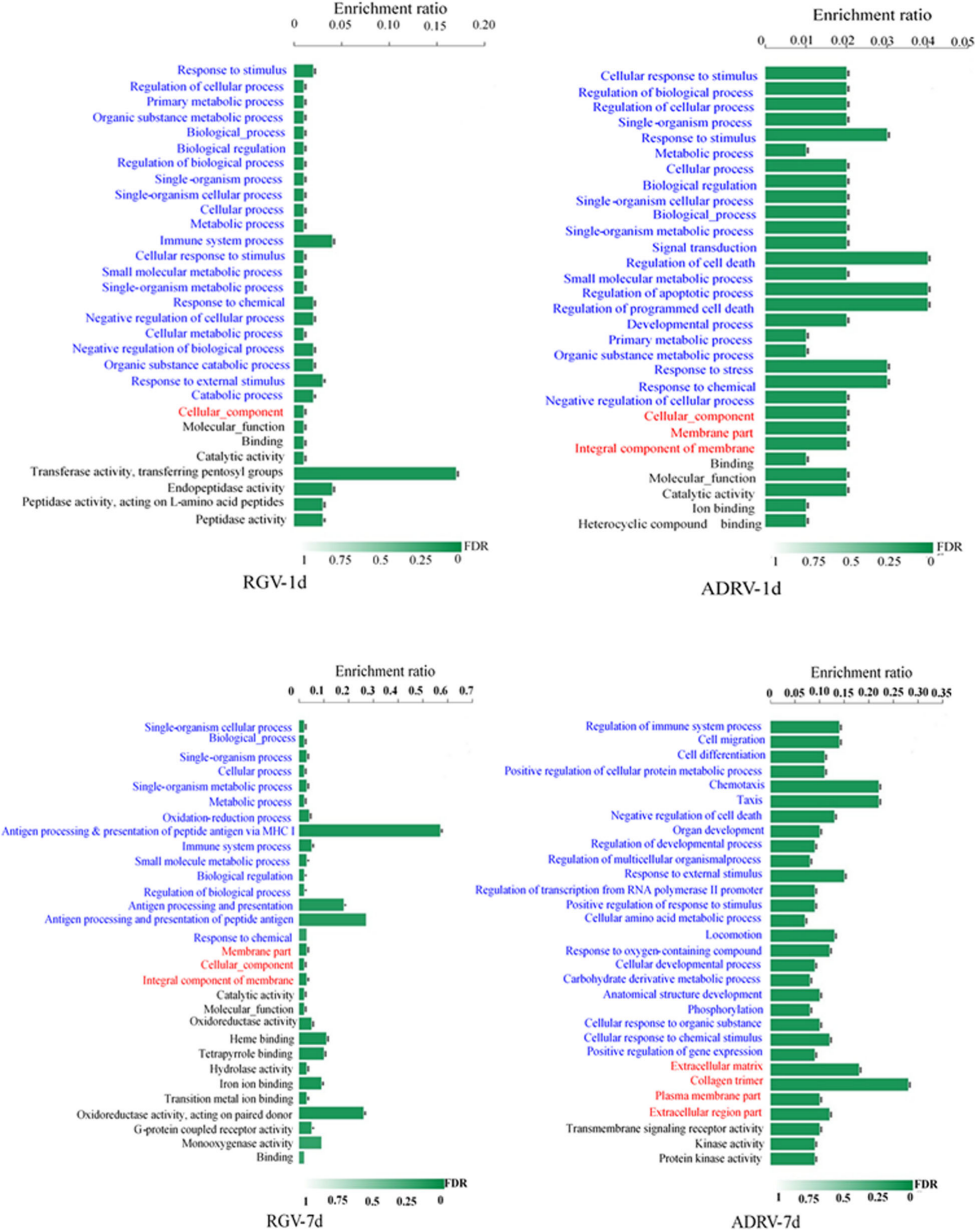
Top 30 significantly enriched Go terms of DEGs (compared with control) in RGV-1d, ADRV-1d, RGV-7d, and ADRV-7d. The Go terms with blue font belonged to biological process (BP), with red font belonged to cellular component (CC), and black font belonged to molecular function (MF). Enrichment ratio was calculated with the formula: Sample number/Background number

DEGs obtained from groups at l day post injection (dpi) were significantly enriched into 58 (RGV) and 85 (ADRV) Go (Gene Ontology) terms respectively. In the top 30 Go terms, both comparisons showed relatively similar responses including “response to stimulus” and “regulation of cellular process” (Fig. 4). There were some distinct top Go terms for the two comparisons. For example, the Go term “Immune system process” was significantly enriched in RGV-1d, while the ADRV-1d contained top Go terms related to cell death including “Regulation of cell death”, “Regulation of apoptotic process”, and “Regulation of programmed cell death”. DEGs from groups at 7 dpi were significantly enriched into 26 (RGV) and 435 (ADRV) Go terms. There were relatively large differences in the top enriched Go terms between RGV-7d and ADRV-7d (Fig. 4). Significantly enriched Go terms focused on biological, cellular, and metabolic process and immune response in RGV-7d. Notably, the Go term “Antigen processing and presentation of peptide antigen via MHC I” was largely enriched in RGV-7d. However, Go terms related to cellular events including “cell migration” and “cell differentiation” were significantly enriched in ADRV-7d besides the Go terms related to stimulus and immune response. These results revealed the differences in host transcriptome responses to two kinds of viruses.

For the top 30 Go terms between RGV infected groups at 1 dpi and 7 dpi, RGV-7d showed more enriched Go terms in antigen processing and presentation, as well as oxidation reduction in the category of biological process. Most of the top 30 Go terms were different between ADRV-1d and ADRV-7d. Detailed information of all the enriched Go terms was collected in Additional file 4: Table S5-S8.

In addition, the results showed that the enrichment ratio had a wide range across different comparisons. The differences among the enrichment ratio were resulted from the differences in sample number, which is the number of enriched DEGs of the Go term. Because the number of DEGs was divergent among the four groups, the scores were different across different comparisons, which reflected the divergent transcriptome responses under different conditions.

DEGs of RGV-1d, RGV-7d, ADRV-1d, and ADRV-7d were significantly enriched into 13, 8, 15, and 20 KEGG (Kyoto Encyclopedia of Genes and Genomes) pathways respectively (Table 2). 10 of the significantly enriched pathways including “cytokine-cytokine receptor interaction”, “malaria”, “hematopoietic cell lineage”, “transcriptional misregulation in cancer”, “Jak-STAT signaling pathway”, “TNF signaling pathway”, “rheumatoid arthritis”, “AGE-RAGE signaling pathway in diabetic complications”, “toll-like receptor signaling pathway”, and “legionellosis” were identical between RGV and ADRV infected groups at 1 dpi. However, RGV-1d contained the enriched pathway “NF-kappa B signaling pathway” and ADRV-1d had the enriched pathway “Antigen processing and presentation” and “Complement and coagulation cascades”. The pathways “cytokine-cytokine receptor interaction”, “hematopoietic cell lineage”, “natural killer cell mediated cytotoxicity”, and “cell adhesion molecules” were possessed in top enriched pathways between RGV and ADRV infected groups at 7 dpi. The pathway “Antigen processing and presentation” was enriched in RGV-7d but not ADRV-7d. The enriched pathway analysis revealed the differences in immune related pathways in host responses to two viruses invading.

**Table 2:** Significantly enriched KEGG pathways in CGS under interspecies and natural pathogen infection

RGV-1d		ADRV-1d		RGV-7d		ADRV-7d	
KEGG	*p*-values	KEGG	*P*-values	KEGG	*P*-values	KEGG	*P*-values
**TNF signaling pathway**	6.76E-05	**Cytokine-cytokine receptor interaction**	3.02E-08	**Hematopoietic cell lineage**	0.006444	**Cytokine-cytokine receptor interaction**	1.78E-09
**Cytokine-cytokine receptor interaction**	0.000262	**Malaria**	6.89E-05	**Cytokine-cytokine receptor interaction**	0.006444	**Hematopoietic cell lineage**	6.46E-07
**Malaria**	0.001122	**Hematopoietic cell lineage**	0.000204	Antigen processing and presentation	0.006444	NF-kappa B signaling pathway	5.46E-05
**Rheumatoid arthritis**	0.002056	**Transcriptional misregulation in cancer**	0.000258	**Cell adhesion molecules**	0.006444	Malaria	0.000397
**Hematopoietic cell lineage**	0.002056	**Jak-STAT signaling pathway**	0.000330	Primary immunodeficiency	0.006444	Rheumatoid arthritis	0.001960
**Jak-STAT signaling pathway**	0.003073	**TNF signaling pathway**	0.000506	**Natural killer cell mediated cytotoxicity**	0.008181	Jak-STAT signaling pathway	0.004337
**Transcriptional misregulation in cancer**	0.020116	Antigen processi and presentation	0.000970	Graft-versus-host disease	0.020223	AGE-RAGE signaling pathway in diabetic complications	0.007612
**Legionellosis**	0.021996	**Rheumatoid arthritis**	0.001018	Glutathione metabolism	0.036874	Complement and coagulation cascades	0.008071
Adipocytokine signaling pathway	0.029942	**AGE-RAGE signaling pathway in diabetic complications**	0.004286			**Natural killer cell mediated cytotoxicity**	0.013214
Osteoclast differentiation	0.029942	African trypanosomiasis	0.016042			Measles	0.019046
NF-kappa B signaling pathway	0.030993	**Toll-like receptor signaling pathway**	0.025041			**Cell adhesion molecules**	0.020083
**Toll-like receptor signaling pathway**	0.030993	**Legionellosis**	0.025041			Transcriptional misregulation in cancer	0.022477
**AGE-RAGE signaling pathway in diabetic complications**	0.030993	Leishmaniasis	0.026544			Leishmaniasis	0.022477
		Herpes simplex infection	0.036416			Hepatitis C	0.022477
		Complement and coagulation cascades	0.038546			Intestinal immune network for IgA production	0.022477
						RIG-I-like receptor signaling pathway	0.026331
						Allograft rejection	0.033481
						*Staphylococcus aureus* infection	0.038645
						Toll-like receptor signaling pathway	0.046413
						Epithelial cell signaling in *Helicobacter pylori* infection	0.047717

Only 8 KEGG pathways were significantly enriched in RGV-7d. The same KEGG pathways between two groups at the same time points were indicated with **bold** font

For the top enriched KEGG pathways between RGV infected groups at 1 dpi and 7 dpi, “hematopoietic cell lineage” and “cytokine-cytokine receptor interaction” were the 2 common pathways for both comparisons. 10 significantly enriched pathways were identical between ADRV infected groups at 1 dpi and 7 dpi, including “cytokine-cytokine receptor interaction”, “hematopoietic cell lineage”, “malaria”, “transcriptional misregulation in cancer”, “rheumatoid arthritis”, “Jak-STAT signaling pathway”, “AGE-RAGE signaling pathway in diabetic complications”, “toll-like receptor signaling pathway”, “leishmaniasis”, and “complement and coagulation cascades” (Table 2). Detailed information of all the enriched KEGG pathways was collected in Additional file 5: Table S9-S12. Obviously, the host responses to specific virus were divergent at different time periods.

### Immune related DEGs and pathways involved in interspecies and natural infection

The DEGs related to immune pathways were selected for further analysis. For RGV-1d, 31 unigenes were identified as DEGs related to immune pathway with 30 up-regulated, while 52 unigenes (37 up-regulated) were identified in ADRV-1d. All of DEGs in RGV-1d except 3 were also found as DEGs in ADRV-1d. For RGV-7d, there were 61 DEGs (34 up-regulated) related to immune pathway, while 166 DEGs (109 up-regulated) were identified in ADRV-7d. The top DEGs were shown in the Table 3. 3 unigenes with homologies to Antigen peptide transporter 2 (TAP2), a member of the superfamily of ATP-binding cassette (ABC) transporters, were highly up-regulated in all comparisons. It indicated that some components involved in antigen processing and presentation were activated by both viruses all the time. Detailed information of all DEGs related to immune pathway was shown in Additional file 6: Table S13-S16.

**Table 3:** Top 10 up- and down-regulated DEGs of CGS related to immune response. Only 1 down-regulated DEG was found in RGV-1d

	RGV-1d		ADRV-1d		RGV-7d		ADRV-7d	
	Unigene ID (Gene symbol)	log_2_FC	Unigene ID (Gene symbol)	log_2_FC	Unigene ID (Gene symbol)	log_2_FC	Unigene ID (Gene symbol)	log_2_FC
Up	c175700_g1 (ABCB3)	6.58	c175700_g1 (ABCB3)	6.69	c175700_g1 (ABCB3)	8.2	c250155_g1 (IFNα)	6.92
	c175700_g11 (ABCB3)	6.45	c175700_g11 (ABCB3)	6.63	c175700_g11 (ABCB3)	7.98	c175700_g1 (ABCB3)	6.32
	c175700_g3 (ABCB3)	5.85	c175700_g3 (ABCB3)	6.48	c175700_g8 (ABCB3)	7.71	c175700_g11 (ABCB3)	6
	c145887_g1 (IL1R2)	4.41	c165419_g4 (IL6)	4.38	c175700_g3 (ABCB3)	7.51	c255707_g1 (MHCI-2)	5.96
	c227211_g1 (CCL3)	3.49	c171455_g1 (CSF3)	4.34	c255707_g1 (MHCI-2)	6.73	c175700_g3 (ABCB3)	5.26
	c165419_g4 (IL6)	3.44	c145887_g1 (IL1R2)	4.22	c178187_g8 (MYLK)	3.46	c165419_g4 (IL6)	5.02
	c171455_g1 (CSF3)	3.25	c130377_g1 (FOS)	3.85	c6198_g1 (CD142)	2.36	c171455_g1 (CSF3)	4.85
	c182194_g4 (CD45)	3.07	c119826_g1 (IL8)	3.32	c171449_g1 (A2M)	2.27	c227211_g1 (CCL3)	4.85
	c141851_g1 (TLR5)	3.02	c141851_g1 (TLR5)	3.07	c131872_g1 (CFB)	2.17	c180346_g1 (ISG15)	4.42
	c6198_g1 (CD142)	2.68	c6198_g1 (CD142)	2.91	c145563_g1 (PAK1)	1.96	c119826_g1 (IL8)	4.06
Down	c140191_g3 (ARRB)	−1.66	c140191_g3 (ARRB)	−1.51	c172210_g1 (COL3A)	−2.81	c166111_g1 (CD23)	−3.41
			c152516_g3 (TNFSF10)	−1.48	c175190_g1 (CD72)	−2.25	c140191_g3 (ARRB)	−3.12
			c148919_g1 (ITGA6)	−1.43	c140191_g3 (ARRB)	−2.21	c172210_g1 (COL3A)	−2.55
			c174569_g4 (CCR8)	− 1.43	c157255_g1 (CD46)	− 1.83	c161191_g1 (FLT3)	−2.1
			c100947_g1 (CCL3)	−1.35	c174569_g4 (CCR8)	−1.77	c181051_g1 (TLR8)	−2.1
			c62459_g1 (C3)	−1.35	c50156_g1 (CD8B)	−1.64	c50156_g1 (CD8B)	−2.07
			c161191_g1 (FLT3)	−1.21	c204796_g1 (IGH)	−1.62	c174659_g4 (GZMB)	−1.99
			c161569_g1 (VCAM1)	−1.18	c96738_g1 (CXCR3)	−1.45	c256484_g1 (RPB10)	−1.87
			c169679_g1 (PRF1)	−1.18	c178769_g7 (LCK)	−1.34	c147656_g1 (ITGB7)	−1.82
			c171198_g1 (MHCI-1)	−1.16	c181447_g2 (CD8A)	−1.34	c4388_g1 (CFL)	−1.72

A total of 7 immune pathways were found significantly enriched by KEGG enrichment analysis of these DEGs. RGV-1d and ADRV-1d both had enriched pathways “hematopoietic cell lineage (HCL)” and “toll-like receptor signaling pathway (TLR)”. However, the pathways “complement and coagulation cascades (CCC)” and “antigen processing and presentation (APP)” were only found in ADRV-1d. For RGV-7d and ADRV-7d, the 2 pathways “HCL” and “natural killer cell mediated cytotoxicity (NKC)” were enriched in both comparisons, while the pathways “TLR”, “CCC”, “intestinal immune network for IgA production (III)”, and “RIG-I-like receptor signaling pathway (RIG)” were enriched in ADRV-7d, and the pathway “APP” was enriched in RGV-7d (Table 1). It was found that the pathway “HCL” was significantly enriched in all the four comparisons, and the pathway “CCC” was only significantly enriched in ADRV groups.

### Regulation of specific host immune related genes by natural pathogen infection

**Fig. 5:**
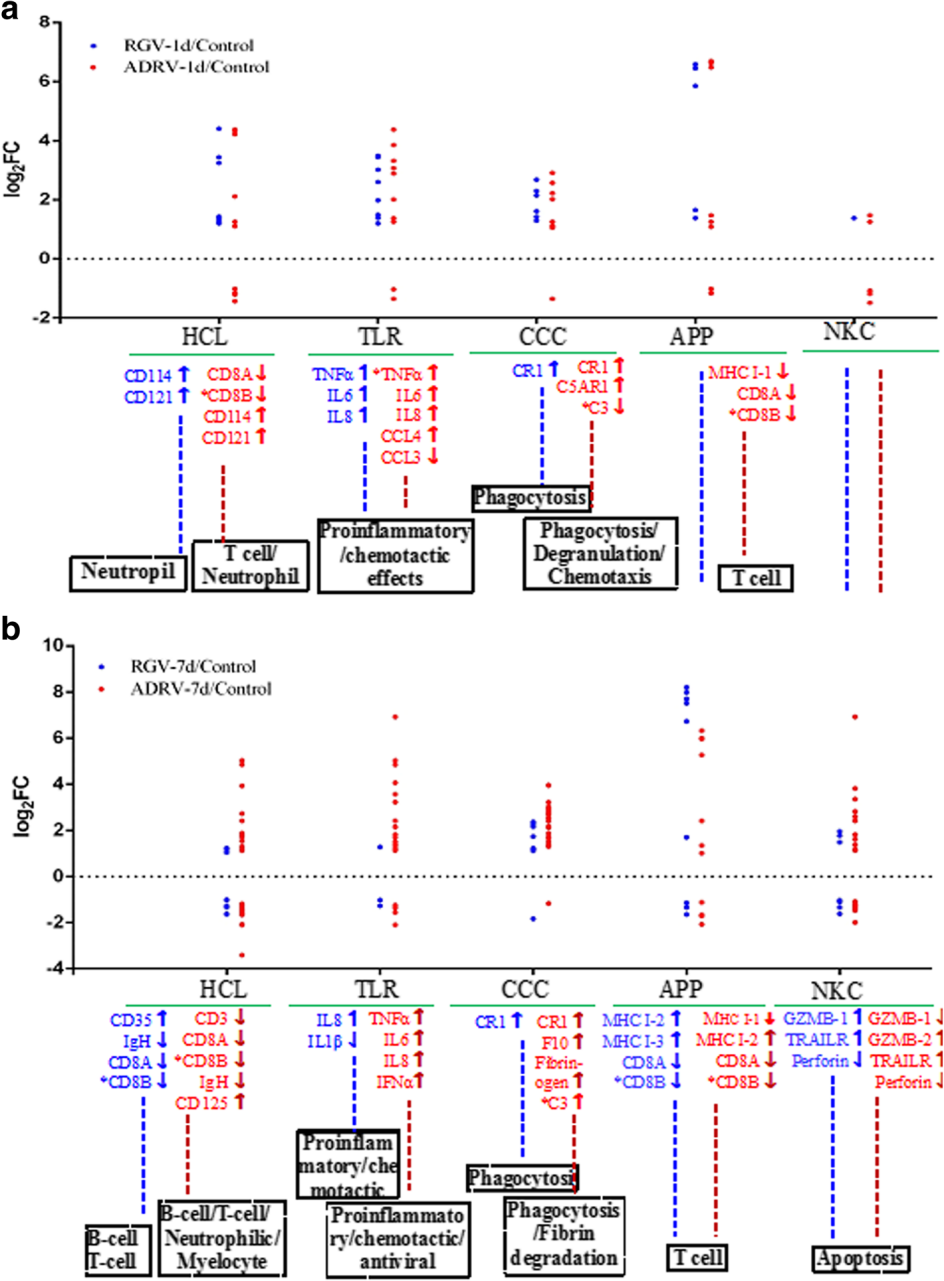
Scatter plots of expression patterns of specific DEGs. DEGs in significantly enriched KEGG pathways related to immune response at 1 dpi (**a**) and 7 dpi (**b**) were selected for analysis. HCL: Hematopoietic cell lineage; TLR: Toll-like receptor signaling pathway; CCC: Complement and coagulation cascades; APP: Antigen processing and presentation; NKC: Natural killer cell mediated cytotoxicity. Each spot indicates a DEG. Key DEGs between ADRV and RGV groups were shown below the scatter plots with different font colors (blue font indicates RGV group and red font indicates ADRV group). Up-regulated DEGs were marked with “↑” and down-regulated DEGs were shown with “↓”. The genes that were detected by RT-qPCR were marked with “*”. Possible functions or targets of the pathway were indicated in dashed box

DEGs related to the 5 significantly enriched immune pathways (HCL, TLR, CCC, APP, and NKC) that were found in at least 2 groups were selected for further investigation. The significantly enriched DEGs were mapped to specific positions of the 5 pathways. As shown in Fig. 5 and Additional file 7-S17-S21, compared with RGV, ADRV infection alone induced the up-regulation of CR1 and C5AR1 at 1 dpi, which was related to phagocytosis and degranulation, but the down-regulation of MHC I-1 (Unigene 1 of MHC I), CD8A, and CD8B, which were key components in antigen processing and presentation. Both RGV and ADRV infection induced the up-regulation of cytokines at 1 dpi, such as IL6, IL8, TNFα, and CD114, which could benefit the neutrophil formation and proinflammatory and chemotactic effects. For samples at 7 dpi, RGV infection alone induced the up-regulation of CD35, GZMB-1, MHC I-3 (Unigene 3 of MHC I) and down-regulation of IL1β. Besides up-regulation of genes related to neutrophil formation, proinflammatory and chemotactic effects, and phagocytosis, ADRV infection induced up-regulation of genes related to fibrin degradation (F5 and F10) and antiviral effects (IFNα), and down-regulation of MHC I-1 compared with RGV. In addition, RGV and ADRV infection induced the up-regulation of TRAILR and down-regulation of perforin (related to apoptosis), up-regulation of MHC I-2 (Unigene 2 of MHC I) and the down-regulation of IgH and CD8 (related to B cell and T cell).

**Fig. 6:**
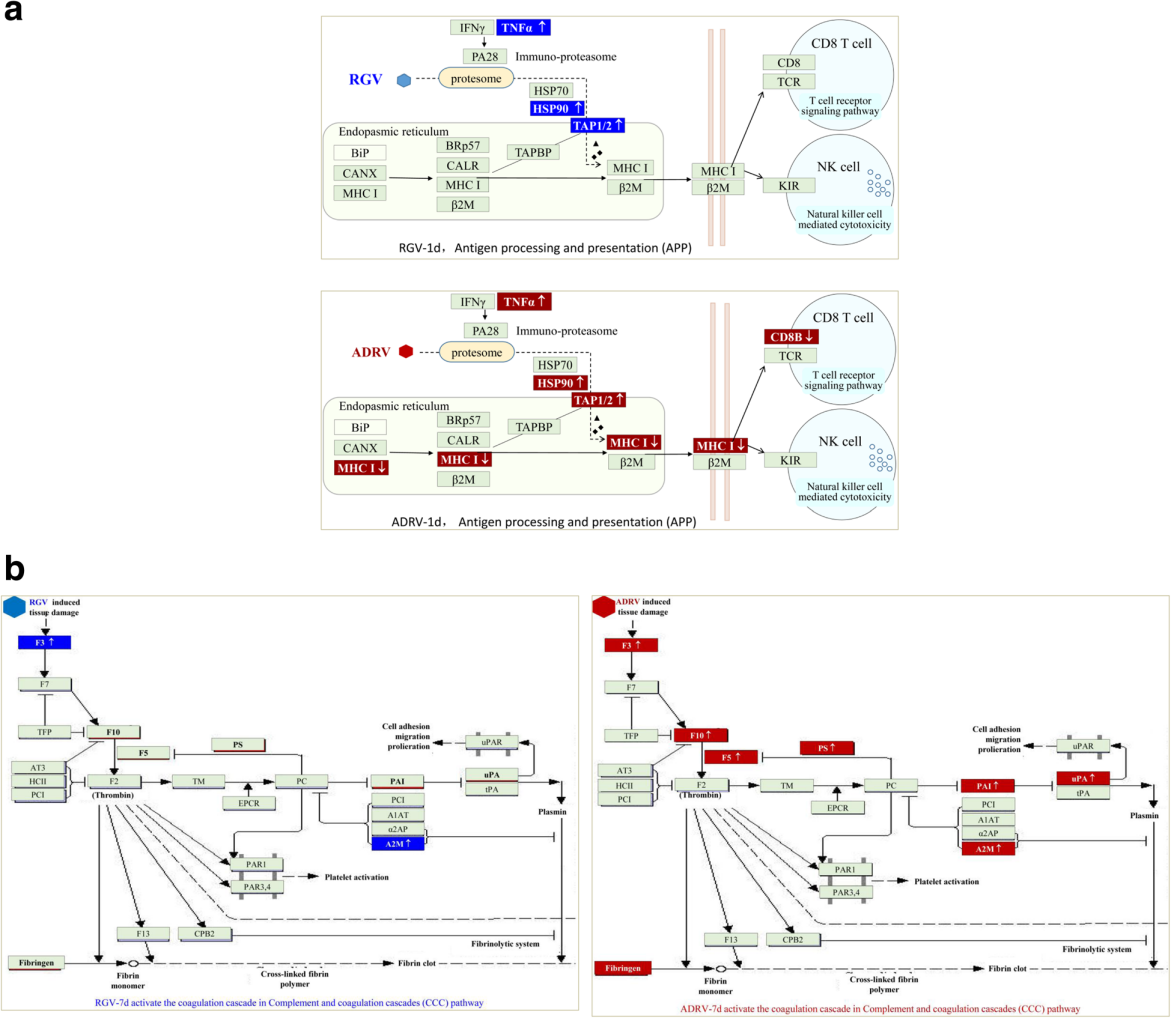
Significantly enriched DEGs in specific KEGG pathways. **a** MHC class I pathway of the KEGG pathway “Antigen processing and presentation” at 1 dpi. **b** coagulation cascades of the KEGG pathway “Complement and coagulation cascades” at 7 dpi. Enriched DEGs of the present study were marked with blue color (RGV) and red color (ADRV). Up- and down-regulated DEGs were marked with “↑” and “↓”, respectively

The MHC class I pathway and Coagulation cascades were further selected from KEGG pathways. As shown in Fig. 6a, RGV infection induced the up-regulation of TNFα, HSP90, and TAP1/2 at 1 dpi. However, ADRV infection induced the down-regulation of MHC I-1 and CD8 (A, B) besides the up-regulation of the genes same as RGV infection. The phenomenon revealed that this pathway was not significantly changed in RGV-1d, because the key downstream molecules MHC I and CD8 were not DEGs in this group. But ADRV activated this pathway with inhibition of MHC I and CD8 expression. For genes related to Coagulation cascades, only 2 genes up-regulated in RGV-7d, while 8 genes up-regulated in ADRV-7d including F10, F5, and Fibrinogen (Fig. 6b). It was consistent with the fact that no hemorrhage symptom was observed in RGV infected CGS compared with ADRV.

### Expression of representative virus genes and host DEGs

**Fig. 7:**
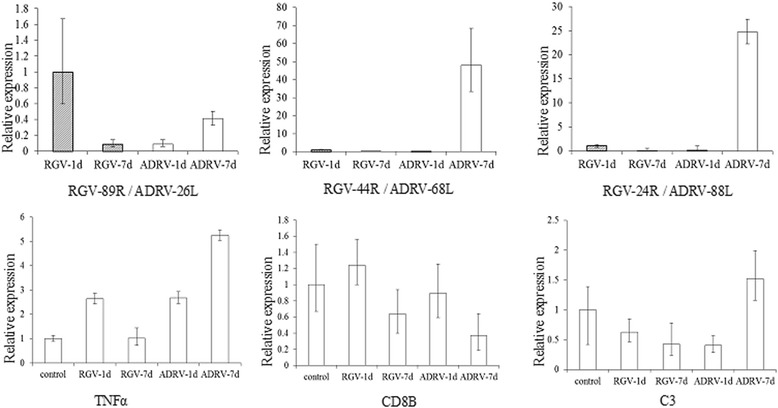
Experimental detection of expression of viral genes and host DEGs by RT-qPCR. Three genes from each virus and three host DEGs were selected for RT-qPCR. Expression levels of viral genes in RGV-1d sample and host genes in control sample were served as 1 in RT-qPCR analysis respectively. The primers and unigene IDs were shown in Additional file 8: Table S22

The selected virus genes, RGV-89R and ADRV-26 L (homologues of ranavirus immediately early protein 18, ICP18), RGV-44R and ADRV-68 L, and RGV-24R and ADRV-88 L, were detected in corresponding samples infected by RGV or ADRV, although expression level in some samples was very low. The expression level of detected viral genes was higher in RGV-1d than that in ADRV-1d, but it was lower in RGV-7d than that in ADRV-7d. RGV-89R (homologue of ICP18) had the highest expression level in RGV-1d among the four groups (especially compared with ADRV 26 L), while ADRV 68 L and 88 L had the highest expression levels in ADRV-7d (compared with its RGV homologues). Expression of host genes (TNFα, CD8B, and C3) was also detected in all samples. TNFα is an important cell signaling protein involved in the pathway “HCL”, “TLR”, “APP”, and “NKC”. CD8B is mainly expressed on the surface of cytotoxic T cell. C3 has a central role in the complement system. Expression of TNFα was largely up-regulated in RGV-1d, ADRV-1d, and ADRV-7d, while the expression of CD8B was down-regulated in RGV-7d, ADRV-1d, and ADRV-7d. The up-regulation of genes related to “complement and coagulation cascades” (such as C3) at ADRV-7d were also revealed (Fig. 7).

## Discussion

Aquaculture in China has been believed to be a major contribution to the world [10]. But its development has been hampered by viral diseases. As members belonging to one of the most primitive orders of the urodele amphibians, CGS has high academic values in researches about evolution and biodiversity [37]. Besides, it has been cultured in different regions of China as part of the aquaculture industry. However, it is easy to suffer from ranaviruses, leading to mass mortalities [12, 32, 40], which could be related to the wide host range of ranaviruses [15], and the interactions with different ranaviruses in water environment. Whether the interspecies infection occurred in CGS and the virus-host interactions in interspecies infection still remains unknown. Transcriptome contains the global information of RNAs (often mRNA) in specific organisms under a given time, which enables a genome-wide survey of interested genes and gene-networks, has been widely used in biology, medicine, agriculture, and so on [41–43]. In the present study, we tried to explore the overall dynamic changes in large DNA virus interspecies infection by using this tool. The different virus replication rates and variant host responses were revealed.

Paired reads covering virus gene and RT-qPCR showed that the replication and gene expression trends of the two viruses were different. The number of virus genes with paired reads and gene expression levels of RGV-1d were all higher than that of ADRV-1d, which suggested the interspecies pathogen replicated faster than natural pathogen at the early stage of infection. This phenomenon, as far as we know, is infrequent in DNA virus interspecies infection. The rapid replication may be an inherent character of RGV, which may be used by DNA virus as a strategy for interspecies infection. The virus replicated rapidly immediately after entry into host cells to get time and space before the unprepared host immune system. In contrast, ADRV may have adapted to CGS under long-term virus-host interactions. It replicated in an unruffled manner. Different viruses may adopt divergent strategies to facilitate their survival. It was easier for RNA virus such as Influenza A virus to adapt and replicate in host because of more frequent mutations and reassortments [44]. However, mutations were fewer in double-stranded DNA viruses than in RNA virus. The high replication rate may be another strategy in large DNA virus infection.

The number of DEGs and immune related pathways of RGV infected group were lower than that of ADRV group, which indicated the host transcriptome responses to RGV was relatively weaker than ADRV. The other interesting phenomenon was that the host responses in RGV-1d were lower than ADRV-1d, which was inconsistent with the virus gene expression. The interspecies pathogen had higher gene expression level, but induced lower host responses than the natural pathogen. Avoiding the overstimulation of host responses could be a strategy for interspecies infection. The exact mechanisms of interactions between RGV and the host need further study. In contrast, different ways were employed by the natural pathogen ADRV. ADRV specifically down-regulated the expression of some host genes, such as MHC I-1 and CD8 (A and B) at the early stage of infection, as well as perforin, a key executor of cytotoxic lymphocytes [45]. MHC class I molecules could present cytosol peptides to CD8 positive (CD8^+^) cytotoxic T cells [46]. CD8^+^ T cells are crucial components of cell-mediated immune responses for providing protection against viral infections [47]. This phenomenon could explain the differences between virus replication *in vitro* and *in vivo*, for the T cells are lacked in cultured GSTC cells. Nevertheless, ADRV induced a more intense response in host though inhibition of some immune related genes. RGV avoided to over-stimulating host immune system at the early stage of infection, but strong immune responses were activated with the time went on because more immune related pathway such as APP and NKC were significantly enriched in RGV-7d. Based on these observations, we speculate that different outcomes for the two viruses may be gained after a period of infection. RGV may be eliminated or released from CGS and infect its natural host frog again. It also could achieve a balance with CGS and persist in it. The other possibility for RGV is that it evolved and adapted to CGS, and then interspecies transmission occurs. RGV might exist for a long time in CGS after the early infection, for the viral reads appeared at 7 dpi, which would facilitate the virus adaptation and the exchange of genetic materials between different viruses [48]. Different host susceptibility and longtime persistence have been reported in other ranaviruses [27, 49–51]. The possibilities of RGV during its infection need to be proved in the future.

Expression level of DEGs in the pathway “complement and coagulation cascades” was divergent between RGV-7d and ADRV-7d. The complement system is an important part of the innate immunity that contains at least 35 or more plasma proteins and cell surface receptors/regulators [52]. This system could recognize and eliminate invaders such as viruses. On the other hand, viruses have also developed multiple strategies against the complement system [53]. At later stage of infection, the system was activated by the abundant replication of viruses. Fewer up-regulated complement system genes of RGV-7d than that of ADRV-7d was in accordance with the fact that the expression level of RGV gene was lower than that of ADRV at 7 dpi. It has been reported that hyperactivity of the complement system can lead to endothelial and blood cell damage, which would give rise to hemolysis, platelet activation and aggregation, and prothrombotic and inflammatory changes [54, 55]. Thus, the up-regulation of genes in complement system at later stage of ADRV infection may be involved in the systemic hemorrhage of diseased CGS, which was also reflected by the up-regulation of genes in “Coagulation cascades”.

Innate and adaptive immune system have been reported to involve in ranavirus-host interactions [56–60]. Although high genome sequence colinearity and similarity were found between RGV and ADRV [32], the differences in their gene expression and amino acids sequences might result in different immune responses *in vivo*. It has been reported that cytomegalovirus could inhibit antigen presentation to T cells [61]. The viral host shutoff proteins of some herpesviruses could degrade host MHC class I mRNA to interfere the MHC class I-mediated peptide presentation [62]. Further study about the interactions between RGV/ADRV and MHC I/CD8^+^ T cells would benefit the understanding of interspecies infection. The polymorphism of MHC I and functions of its isoforms should also be investigated. Some proteins of ranaviruses have been identified as antagonist to mediate host immunity, such as homologs of eukaryotic translation initiation factor 2 alpha (eIF-2α) [18, 63, 64]. The finding that ADRV encodes a full length eIF2α-like protein (ORF84L) but RGV encodes a truncated homolog (ORF28R) enhanced our interest to explore the different virus-host interactions in the future.

## Conclusions

**Fig. 8:**
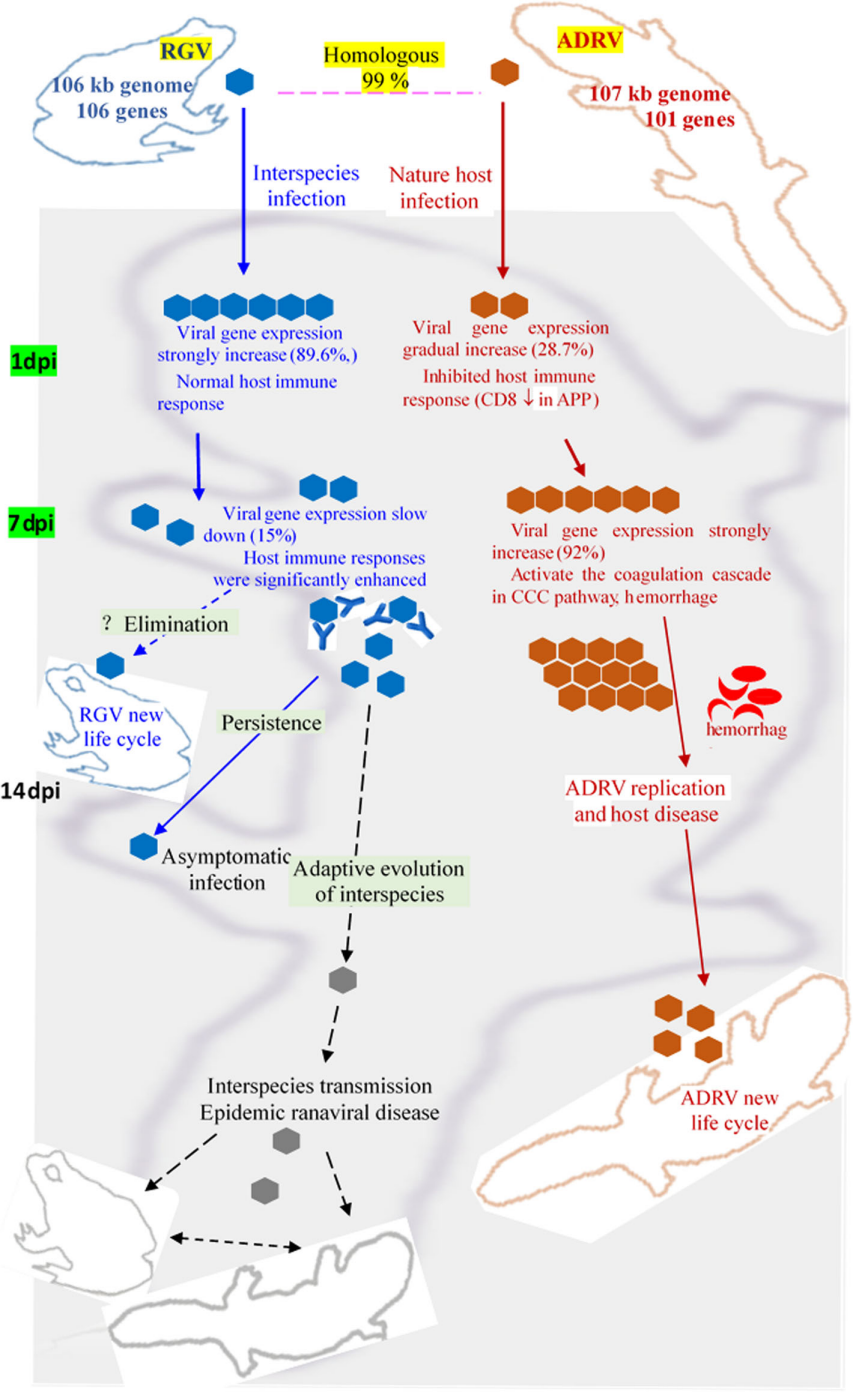
Illustration of CGS under interspecies and natural pathogen infection. Events occurred in RGV infection were shown in blue color and that related to ADRV was shown in red color. The events that proved in the present study were indicated by solid arrows, whereas that might occur were indicated by dotted arrows

In conclusion, different strategies were employed by the two viruses in interspecies and natural infection of CGS, which then induced divergent host responses in transcriptional levels. RGV replicated rapidly and avoided to over-stimulating host immune system in interspecies infection. In contrast, the natural pathogen ADRV had a moderate replication rate with the inhibition of pivotal components of host immune system. However, stronger immune responses were activated in interspecies infection as time went on, which then lead to different fate for RGV. Abundant proliferation of ADRV occurred because of the inhibition of host immunity, which then led to stronger host responses. After a period of infection, different outcomes for the two viruses may be gained (Fig. 8).

## Methods

### Viruses and interspecies infection *in vitro* and *in vivo*

RGV that isolated from diseased pig frog *Rana grylio* [36] was used as non-natural pathogen for interspecies infection. At the same time, ADRV that isolated from CGS [32] was used for comparison as natural pathogen in the present study.

For detection of interspecies infection *in vitro*, One-step virus growth curves of RGV were performed in CGS thymus cell (GSTC) line as described previously [39]. Briefly, GSTC cells were grown in 96-well plates and infected with RGV at an m.o.i of 0.1. The cells were harvested at various intervals (0, 1d, 2d, 3d, 4d, 5d, and 6d) and titrated on duplicate monolayers of GSTC cells. The one-step virus growth curve of ADRV was also performed under same conditions.

Ultrastructural observation was performed as described by Huang et al. [28]. GSTC cells were infected with RGV and ADRV respectively. At 1 dpi, the cells were harvested and collected by centrifugation. The pellets were fixed with 2.5% glutaraldehyde, followed by 1% OsO4. After dehydrated with ethanol, the cells were embedded in Epon-812, and then were sectioned and stained. The ultra-thin sections were examined with a JEM-1230 electron microscopy at 100 kV and micrographs were taken by CCD camera.

For interspecies infection *in vivo*, health cultured CGSs of 95 g mean mass were obtained from a farm in Jiangxi, China, and acclimated in aerated dechlorinated water at 22 °C for 2 weeks before initiation of the experiment. The animals were fed with commercial feed and water was replaced daily.

After 2 weeks acclimation, health CGSs were randomly selected to perform interspecies infection. Six CGSs were intraperitoneally injected with 200 μl RGV (6 × 10^6^ TCID_50_) respectively and the other six CGSs were injected with 200 μl ADRV (6 × 10^6^ TCID_50_) under same operations. Another three were injected with 200 μl PBS each as control.

At 1 day post-injection (dpi), spleens were collected from three individuals of RGV injected CGSs and denoted as RGV-1d. Simultaneously, spleens were collected from three individuals of ADRV and PBS injected CGSs and recorded as ADRV-1d and Control, respectively. At 7 dpi, spleens were collected from RGV and ADRV injected CGSs as described above and recorded as RGV-7d and ADRV-7d, respectively.

All surgery was performed under benzocaine anesthesia, and all efforts were made to minimize suffering. Animals were sacrificed by intraperitoneal injection of sodium pentobarbital.

### RNA isolation, library construction and sequencing

Total RNA of collected spleens from 15 individuals (group RGV-1d, RGV-7d, ADRV-1d, ADRV-7d, and Control; *n* = 3) were extracted with TRizol reagent (Invitrogen, USA) following the manufacturer’s protocol. RNA integrity, purity, and concentration were determined by electrophoresis and the NanoDrop 2000 spectrophotometer (ThermoFisher, USA). RNA samples with high quality were used in library construction. Libraries were constructed using the TruseqTM RNA sample prep kit (Illumina, USA) following the manufacturer’s protocol. Briefly, mRNA was purified by using poly-T oligo-attached magnetic beads and fragmented with fragmentation buffer. After first and second cDNA synthesis, the cohesive ends of cDNA were repaired and then adenosines were added to the 3′ ends. Adapters were ligated to the cDNA and then cDNA fragments were enriched by PCR. PCR products were purified using Certified Low Range Ultra Agarose (Bio-Rad, USA) and quantified with TBS380 Picogreen (Invitrogen, USA). Libraries were sequenced on Illumina HiSeq platform using HiSeq 4000 SBS Kit (Illumina, USA) and generated raw data reads.

### Data analysis

The raw data reads were processed using software SeqPrep and Sickle to remove adapter, poly-N and poor quality data. The resulted data was clean data (clean reads) and its Q20, Q30, and GC contents were calculated to evaluate the data quality. De novo transcriptome assembly was performed using Trinity software [65] based on the clean data. Unigenes acquired from the assembly were annotated to NR, Pfam, String, Swissprot, and KEGG databases by Trinity and BlastX software (E-value cut-off of 1.0E-5).

Expression levels of unigenes were counted using RSEM software [66]. The results (Additional file 8: Table S22) were presented as numbers of fragments per kilobase of transcript per million fragments sequenced (FPKM) and used for unigene expression analysis. The clean reads were mapped to virus genome using bowtie2 [67]. Number of fragments (paired reads) covering each virus gene were counted using featureCounts [68].

### Differential expression analysis

Differential expression analysis between two groups (virus infected group compared to control) was performed using edgeR package [69] base on the unigenes obtained from Trinity assembly and expression levels from RSEM software. Genes with FDR < 0.05 and |log_2_Fold Change| (|log_2_FC|) ≥ 1 were assigned as differentially expressed genes (DEGs). Venn diagram was created using VennDiagram [70] to show the quantitative distribution of DEGs in different comparisons. Gene ontology (GO) annotation was performed using blast2go software [71] to classify the DEGs. Pathway analysis was performed using the Kyoto Encyclopedia of Genes and Genomes (KEGG) database [72]. The enrichment analyses of Go terms and KEGG pathways were performed using Goatools [73] and KOBAS [74] based on Fisher’s exact test, respectively. Multiple corrections including Bonferroni, Holm, Sidak, and false discovery rate were used for correcting the *P*-value as described by Lu et al. [75]. Targets with corrected P-value ≤0.05 were assigned as significantly enriched.

### Experimental detection of virus genes and host DEGs by RT-qPCR

Three pairs of virus genes (each pair were homologous: RGV-89R and ADRV-26 L, RGV-44R and ADRV-68 L, and RGV-24R and ADRV-88 L) and three host genes (TNFα, CD8B, and C3) were selected for real-time quantitative PCR (RT-qPCR) analysis. Primers used in this assay were listed in Additional file 9: Table S23. RNA from the same samples in RNA-Seq was used as templates. First strand cDNA synthesis was performed using PrimeScript RT reagent Kit with gDNA Eraser (TaKaRa, Japan). RT-qPCR was conducted using a StepOne Real-Time PCR system (The Applied Biosystems, USA). Each RT-qPCR mixture contained 1 μl of cDNA, 12.5 μl of SYBR Premix (2×), 0.5 μl of forward and reverse primers (for each primer), and 10.5 μl of ultrapure water. The RT-qPCR conditions were as follows: 95 °C for 10 min; 40 cycles of 95 °C for 15 s and 60 °C for 1 min; and a melt curve analysis at 95 °C for 15 s, 60 °C for 1 min, and 95 °C for 15 s. The β-actin gene was used as internal control. The mRNA relative expression ratios of the treated group versus that of the control group were calculated by the 2^-ΔΔCT^ method [76].

## Additional files

Additional file 1: **Table S1.** The paired reads covering each gene of ADRV in ADRV-1d and ADRV-7d. **Table S2.** The paired reads covering each gene of RGV in RGV-1d and RGV-7d. GenBank accession number of the virus genome, start and end position of ORF (gene), gene length, counts of each sample were shown. (ZIP 24 kb)

Additional file 2: **Figure S1.** Sequence length distribution of unigenes from all libraries. The smallest, largest, average, N50, and N90 length were shown in the figure. (TIFF 148 kb)

Additional file 3: **Table S3.** Top 10 DEGs in comparison of RGV-1d/control and ADRV-1d/control. **Table S4.** Top 10 DEGs in comparisons of RGV-7d/control and ADRV-7d/control. Unigene IDs of DEGs possessed by two groups (RGV-1d/control and ADRV-1d/control, RGV-7d/control and ADRV-7d/control) were indicated in **bold** font. “-” means no homolog found. (ZIP 19 kb)

Additional file 4: **Table S5.** Detailed information of all the enriched Go terms in RGV-1d/control. **Table S6.** Detailed information of all the enriched Go terms in ADRV-1d/control. **Table S7.** Detailed information of all the enriched Go terms in RGV-7d/control. **Table S8**. Detailed information of all the enriched Go terms in ADRV-7d/control. List of Go terms include the ID and description of Go terms, enrichment ratio, corrected *p*-value, main categories, and enriched DEGsn. (ZIP 339 kb)

Additional file 5: **Table S9.** Detailed information of all the enriched KEGG pathways in RGV-1d/control. **Table S10.** Detailed information of all the enriched KEGG pathways in ADRV-1d/control. **Table S11.** Detailed information of all the enriched KEGG pathways in RGV-7d/control. **Table S12.** Detailed information of all the enriched KEGG pathways in ADRV-7d/control. List of KEGG pathways include pathway name, number of DEGs and unigene IDs enriched in corresponding pathway, corrected p-value, and hyperlink of the pathway. (ZIP 124 kb)

Additional file 6: **Table S13.** Detailed information of DEGs related to immune pathways in RGV-1d/control. **Table S14.** Detailed information of DEGs related to immune pathways in ADRV-1d/control. **Table S15.** Detailed information of DEGs related to immune pathways in RGV-7d/control. **Table S16.** Detailed information of DEGs related to immune pathways in ADRV-7d/control. List of DEGs include the sequence ID, counts and fpkm value, log_2_fold change, p-value, FDR, and annotations in NR, Swissprot, String, KEGG, and Pfam. (ZIP 99 kb)

Additional file 7: Gene expression in representative immune pathways. **Table S17.** Gene expression level in the pathway “Hematopoietic cell lineage”. **Table S18.** Gene expression level in the pathway “Toll-like receptor signaling pathway”. **Table S19.** Gene expression level in the pathway “Complement and coagulation cascades”. **Table S20.** Gene expression level in the pathway “Antigen processing and presentation”. **Table S21.** Gene expression level in the pathway “Natural killer cell mediated cytotoxicity”. The log_2_(fold change) of each DEG assigned to corresponding pathway was shown. (ZIP 45 kb)

Additional file 8: **Table S22.** FPKM of all unigenes. (XLSX 14778 kb)

Additional file 9: **Table S23.** Genes and primers used for RT-qPCR. (DOCX 16 kb)

## Abbreviations

ADRV: *Andrias davidianus* ranavirus
C3: Complement component 3
C5AR1: Complement component 5a receptor 1
CCL3: Chemokine (C-C motif) ligand 3
CD114: Cluster of Differentiation 114
CD35: Cluster of differentiation 35
CD8B: Cluster of differentiation 8
CGS: Chinese giant salamander
CR1: Complement receptor type 1
DEG: Differentially expressed gene
dpi: Day post-injection
eIF-2α: Eukaryotic translation initiation factor 2 alpha
F10: Factor X
F5: Factor V
FLT3: Fms like tyrosine kinase 3
GO: Gene Ontology
GSTC: CGS thymus cell
GZMB: Granzyme B
HSP90: Heat shock protein 90
IFNα: Interferon α
IgH: Immunoglobulin heavy chain
IL1β: Interleukin 1 β
IL6: Interleukin 6
IL8: Interleukin 8
ITGA6: Integrin alpha-6
KEGG: Kyoto Encyclopedia of Genes and Genomes
log_2_FC: log_2_Fold Change
MHC I: Major histocompatibility complex class I
ORF: Open reading frame
RGV: *Rana grylio* virus
RT-qPCR: Real-time quantitative PCR
TAP1/2: Antigen peptide transporter 1/2
TLR1: Toll-like receptor 1
TNFα: Tumor Necrosis Factor α
TRAILR: TRAIL receptor
