# Supplementary figures and images for: Divergent transcriptomic responses underlying the ranaviruses-amphibian interaction processes on interspecies infection of Chinese giant salamander
Source: BMC Genomics. 2018 Mar 20;19:211. doi: 10.1186/s12864-018-4596-y (PMC5861657; doi:10.1186/s12864-018-4596-y)

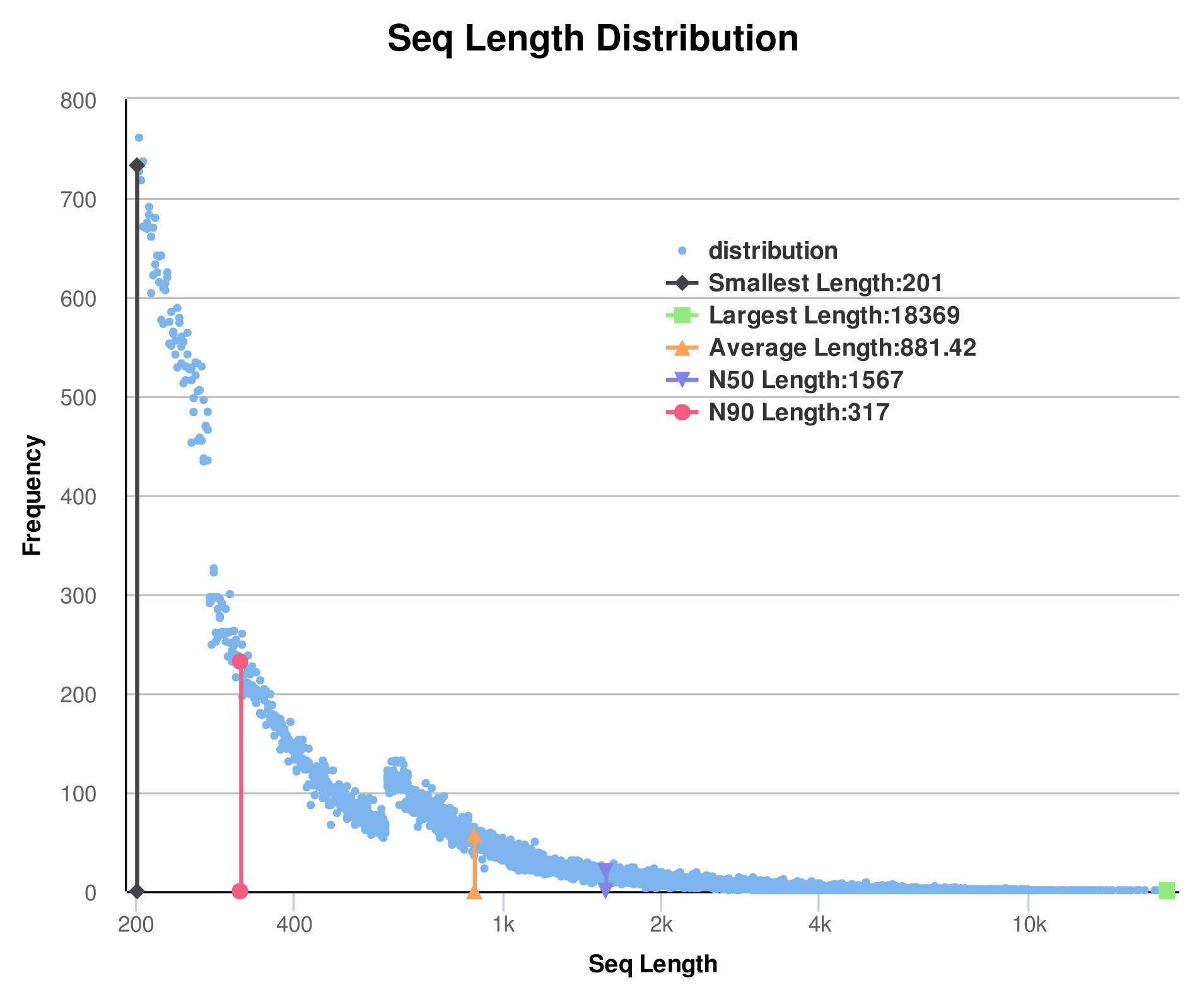

Supplement: Supplementary file 2 — Figure S1. Sequence length distribution of unigenes from all libraries. The smallest, largest, average, N50, and N90 length were shown in the figure. (TIFF 148 kb) [file 12864_2018_4596_MOESM2_ESM.tif]
