# Supplementary material for: Divergent transcriptomic responses underlying the ranaviruses-amphibian interaction processes on interspecies infection of Chinese giant salamander
Source: BMC Genomics. 2018 Mar 20;19:211. doi: 10.1186/s12864-018-4596-y (PMC5861657; doi:10.1186/s12864-018-4596-y)
Supplement: Supplementary file 9 — Table S23. Genes and primers used for RT-qPCR. (DOCX 16 kb) [file 12864_2018_4596_MOESM9_ESM.docx]

| Gene name | Unigene ID | primers |
| --- | --- | --- |
| RGV-89R/ADRV-26L |  | 89R-F: CATTCGCTGTGCCATTCGAC |
|  |  | 89R-R: TGGTGGAGCCCAAGAAGGTC |
| RGV-44R/ADRV-68L |  | 44R-F: TCTGCCTTGAAGAAGACTGCG |
|  |  | 44R-R: CGTGTTTAGCGTGACTGTGCC |
| RGV-24R/ADRV-88L |  | 24R-F: GGGATGGGACCAAACATTACA |
|  |  | 24R-R: TGCATTCGCCAACGGTATCTA |
| TNFα | c292849_g1 | TNFα-F: GGAGCACAAGAAGGCAGAAT |
|  |  | TNFα-R: CTGAGGTCGGCTATGAGGTG |
| CD8B | c50156_g1 | CD8B-F：CGGAGCCCAGATGACGATAC |
|  |  | CD8B-R：AAGTTGGCTGTGGTCGATGT |
| C3 | c62459_g1 | C3-F：TGAGGTGATTGCTGACCGAC |
|  |  | C3-R：TTTACTGACGCCGGCCTATC |

Table S23 Genes and primers used for RT-qPCR
