# Supplementary material for: Divergent transcriptomic responses underlying the ranaviruses-amphibian interaction processes on interspecies infection of Chinese giant salamander
Source: BMC Genomics. 2018 Mar 20;19:211. doi: 10.1186/s12864-018-4596-y (PMC5861657; doi:10.1186/s12864-018-4596-y)
Supplement: Supplementary file 3 — Table S3. Top 10 DEGs in comparison of RGV-1d/control and ADRV-1d/control. Table S4. Top 10 DEGs in comparisons of RGV-7d/control and ADRV-7d/control. Unigene IDs of DEGs possessed by two groups (RGV-1d/control and ADRV-1d/control, RGV-7d/control and ADRV-7d/control) were indicated in bold font. “-” means no homolog found. (ZIP 19 kb) [file 12864_2018_4596_MOESM3_ESM.zip › Table S3 ADRV-1d and RGV-1d.doc]

Table S3 Top 10 DEGs in comparison of RGV-1d/Control and ADRV-1d/Control. Unigene IDs of DEGs possessed by two groups (RGV-1d/Control and ADRV-1d/Control) were indicated in **bold** font. “-” means no homolog found.

|  | RGV-1d/Control | | |  | ADRV-1d/Control | | |  |
| --- | --- | --- | --- | --- | --- | --- | --- | --- |
|  | Gene names | Unigene ID | log_2_FC |  | Gene names | Unigene ID | log_2_FC |  |
| Up | Proteasome subunit beta type-8 | **c90782_g1** | 8.65 |  | Proteasome subunit beta type-8 | **c90782_g1** | 8.83 |  |
|  | Protease, serine, 1 precursor | **c92129_g1** | 8.12 |  | Protease, serine, 1 precursor | **c92129_g1** | 7.74 |  |
|  | - | **c149173_g1** | 7.09 |  | - | **c149173_g1** | 6.79 |  |
|  | ATP-binding cassette sub-family B member 9-like | **c175700_g1** | 6.58 |  | Heat shock 70 kDa protein 12A-like | **c162425_g1** | 6.71 |  |
|  | Heat shock 70 kDa protein 12A-like | **c162425_g1** | 6.49 |  | ATP-binding cassette sub-family B member 9-like | **c175700_g1** | 6.69 |  |
|  | ATP-binding cassette, sub-family B (MDR/TAP), member 3 like 1 | **c175700_g11** | 6.45 |  | ATP-binding cassette, sub-family B (MDR/TAP), member 3 like 1 | **c175700_g11** | 6.63 |  |
|  | - | c151481_g3 | 6.43 |  | ATP-binding cassette sub-family B member 9-like | **c175700_g3** | 6.48 |  |
|  | - | **c124360_g3** | 5.91 |  | Proteasome subunit beta type-9 | c181782_g4 | 6.06 |  |
|  | ATP-binding cassette sub-family B member 9-like | **c175700_g3** | 5.85 |  | - | **c124360_g3** | 6.04 |  |
|  | slc6a14.1 protein [*Xenopus (Silurana) tropicalis*] | c113162_g1 | 5.84 |  | - | c181760_g8 | 5.79 |  |
|  |  |  |  |  |  |  |  |  |
| Down | - | **c178024_g1** | -5.23 |  | - | c168585_g3 | -5.53 |  |
|  | Leucine-, glutamate- and lysine-rich protein 1 | c173302_g4 | -4.55 |  | - | **c178024_g1** | -5.23 |  |
|  | - | **c142005_g1** | -4.4 |  | Olfactory receptor 10A4-like | c157317_g2 | -5.05 |  |
|  | - | c180880_g4 | -4.17 |  | Protein FAM189A2 isoform X2 | c156493_g1 | -4.71 |  |
|  | - | c178559_g2 | -3.98 |  | Hypothetical protein MYCFIDRAFT_54532 | c170384_g1 | -4.49 |  |
|  | uncharacterized protein LOC100494154 | c180880_g1 | -3.7 |  | - | **c142005_g1** | -4.4 |  |
|  | - | c167002_g6 | -3.61 |  | - | c175453_g2 | -4.09 |  |
|  | - | c176442_g8 | -3.48 |  | - | c175453_g1 | -4.05 |  |
|  | uncharacterized protein LOC101732979 | c123057_g1 | -3.41 |  | - | c182066_g3 | -4.02 |  |
|  | - | c105569_g1 | -3.27 |  | - | c157317_g1 | -4 |  |
