# Supplementary material for: Divergent transcriptomic responses underlying the ranaviruses-amphibian interaction processes on interspecies infection of Chinese giant salamander
Source: BMC Genomics. 2018 Mar 20;19:211. doi: 10.1186/s12864-018-4596-y (PMC5861657; doi:10.1186/s12864-018-4596-y)
Supplement: Supplementary file 3 — Table S3. Top 10 DEGs in comparison of RGV-1d/control and ADRV-1d/control. Table S4. Top 10 DEGs in comparisons of RGV-7d/control and ADRV-7d/control. Unigene IDs of DEGs possessed by two groups (RGV-1d/control and ADRV-1d/control, RGV-7d/control and ADRV-7d/control) were indicated in bold font. “-” means no homolog found. (ZIP 19 kb) [file 12864_2018_4596_MOESM3_ESM.zip › Table S4 ADRV-7d and RGV-7d.doc]

|  | RGV-7d/Control | | |  | ADRV-7d/Control | | |
| --- | --- | --- | --- | --- | --- | --- | --- |
|  | Gene names | Unigene ID | log_2_FC |  | Gene names | Unigene ID | log_2_FC |
| up | Proteasome subunit beta type-8 | **c90782_g1** | 10.94 |  | Protease, serine, 1 precursor | **c92129_g1** | 8.82 |
|  | Protease, serine, 1 precursor | **c92129_g1** | 8.36 |  | Proteasome subunit beta type-8 | **c90782_g1** | 8.72 |
|  | - | **c149173_g1** | 8.33 |  | Zinc finger protein RFP-like | c180330_g3 | 7.82 |
|  | Proteasome subunit beta type-9 | c181782_g4 | 8.28 |  | - | c96872_g1 | 7.38 |
|  | ATP-binding cassette sub-family B member 9-like | c175700_g1 | 8.2 |  | Hemopexin-like, partial | c145238_g1 | 7.35 |
|  | - | c308728_g1 | 8.09 |  | - | c175027_g5 | 7.32 |
|  | ATP-binding cassette, sub-family B (MDR/TAP), member 3 like 1 | c175700_g11 | 7.98 |  | Stromelysin-1/2-a | c175110_g1 | 7.25 |
|  | ATP-binding cassette sub-family B member 9-like | c175700_g8 | 7.71 |  | - | **c149173_g1** | 7.24 |
|  | ATP-binding cassette sub-family B member 9-like | c175700_g3 | 7.51 |  | - | c145238_g2 | 7.22 |
|  | pG1 protein | c176209_g1 | 7.34 |  | - | c181149_g2 | 7.08 |
|  |  |  |  |  |  |  |  |
| down | - | c172581_g6 | -8.71 |  | - | **c69083_g1** | -6.53 |
|  | Deleted in malignant brain tumors 1 protein-like | c173012_g1 | -8.03 |  | - | c176647_g1 | -5.73 |
|  | Protein FAM183A | c134701_g1 | -7.66 |  | - | **c205578_g1** | -4.92 |
|  | - | **c69083_g1** | -6.53 |  | - | c177798_g4 | -4.87 |
|  | - | **c205578_g1** | -5.61 |  | - | c177246_g12 | -4.65 |
|  | Zona pellucida-like domain-containing protein 1-like | c150565_g2 | -5.47 |  | Endonuclease reverse transcriptase | c165462_g1 | -4.64 |
|  | - | c25698_g1 | -5.32 |  | Leucine-, glutamate- and lysine-rich protein 1 | c173302_g4 | -4.63 |
|  | - | c164112_g2 | -5.24 |  | - | c180880_g4 | -4.01 |
|  | - | c178024_g1 | -5.23 |  | - | c179487_g2 | -4.01 |
|  | - | c25031_g1 | -5.18 |  | - | c157661_g1 | -3.91 |

Table S4 Top 10 DEGs in comparisons of RGV-7d/Control and ADRV-7d/Control. Unigene IDs of DEGs possessed by two groups (RGV-7d/Control and ADRV-7d/Control) were indicated in **bold** font. “-” means no homolog found.
