# Supplementary material for: Divergent transcriptomic responses underlying the ranaviruses-amphibian interaction processes on interspecies infection of Chinese giant salamander
Source: BMC Genomics. 2018 Mar 20;19:211. doi: 10.1186/s12864-018-4596-y (PMC5861657; doi:10.1186/s12864-018-4596-y)
Supplement: Supplementary file 7 — Gene expression in representative immune pathways. Table S17. Gene expression level in the pathway “Hematopoietic cell lineage”. Table S18. Gene expression level in the pathway “Toll-like receptor signaling pathway”. Table S19. Gene expression level in the pathway “Complement and coagulation cascades”. Table S20. Gene expression level in the pathway “Antigen processing and presentation”. Table S21. Gene expression level in the pathway “Natural killer cell mediated cytotoxicity”. The log2(fold change) of each DEG assigned to corresponding pathway was shown. (ZIP 45 kb) [file 12864_2018_4596_MOESM7_ESM.zip › Table S19 Complement and coagulation cascades.doc]

Table S19 Expression patterns of DEGs in the pathway “Complement and coagulation cascades” in RGV and ADRV infected groups.

| Unigene ID (Gene name) | log_2_FC | | | |
| --- | --- | --- | --- | --- |
|  | RGV-1d/  Control | ADRV-1d/  Control | RGV-7d/  Control | ADRV-7d/  Control |
| c131872_g1(complement factor B, CFB) | 1.61 | 1.11 | 2.17 | 3.94 |
| c171449_g1(A2M) | 2.15 | 2.22 | 2.27 | 2.99 |
| c6198_g1(F3,CD142) | 2.68 | 2.91 | 2.36 | 3.94 |
| c170576_g1(CR1,CD35) | 1.29 | 1.25 |  | 1.72 |
| c169202_g1(CR1,CD35) |  |  | 1.19 | 2.72 |
| c25090_g1(SERPINE1,PAI1) | 1.42 | 2.02 |  | 3.96 |
| c89230_g1(PLAU) | 2.3 | 2.57 |  | 2.39 |
| c179272_g1(C5AR1) |  | 1.05 |  |  |
| c145756_g1(C1S) |  |  | 1.12 | 2.86 |
| c124908_g1(C1R) |  |  |  | 2.51 |
| c166515_g1(SERPING1,C1INH) |  |  | 1.23 | 2.93 |
| c169652_g1(HF1, Factor H) |  |  | 1.74 | 1.38 |
| c62459_g1(C3) |  | -1.35 |  | 1.51 |
| c62459_g2(C3) |  |  |  | 1.32 |
| c292867_g1(C3) |  |  |  | 2.53 |
| c102725_g1(FGA, Fibrinogen α) |  |  |  | 3.01 |
| c144248_g1(FGG, Fibrinogen γ) |  |  |  | 2.68 |
| c161168_g1(F2RL2,PAR3) |  |  |  | 1.66 |
| c172878_g1(PROS1) |  |  |  | 1.31 |
| c172892_g1(F5) |  |  |  | 2.77 |
| c178876_g1(F10) |  |  |  | 1.88 |
| c283791_g1(MBL) |  |  |  | 2.16 |
| c4593_g1(CLU) |  |  |  | 3.21 |
| c182022_g2(C2) |  |  |  | 2.13 |
| c165045_g1(C2) |  |  |  | -1.17 |
| c157255_g1(CD46, MCP) |  |  | -1.83 |  |
